# Cardiac Transplantation following Cobalt Cardiomyopathy from Bilateral Metal-on-Metal Hip Replacements

**DOI:** 10.1155/2022/3373363

**Published:** 2022-06-08

**Authors:** Peter Szedlak, Amrik Virdi, Paul Cacciottolo, Stephen Shepherd, Stephen Pettit, Florian Falter

**Affiliations:** ^1^Royal Papworth Hospital NHS Foundation Trust, Cambridge, UK; ^2^North West Anglia NHS Foundation Trust, Huntingdon, UK; ^3^Barts Health NHS Trust, London, UK

## Abstract

A fifty-two-year-old man underwent heart transplantation at our centre after four years of developing progressive heart failure symptoms due to cobalt toxicity-related cardiomyopathy. Between the ages of forty and forty-two, he underwent bilateral metal-on-metal hip arthroplasties for early onset osteoarthritis. Six years later, he developed increasing fatigue and pericardial effusions. Following a prolonged period of deterioration without a clear cause, the diagnosis of cobalt toxicity-related cardiomyopathy due to cobalt-chromium alloy hip prostheses was eventually made. He underwent bilateral revision hip arthroplasties and was listed for heart transplantation. Metal-on-metal joint replacement is a rare cause of iatrogenic cobalt toxicity. Anaesthetists may encounter patients with unexplained symptoms of heart failure, having a high index of suspicion presenting an opportunity for early diagnosis and intervention before end-stage disease develops.

## 1. Introduction

Cobalt toxicity-related cardiomyopathy (CMP) was first described in 1967, in a case series of 30 patients presenting with heart failure, which was eventually traced back to the ingestion of large amounts of cobalt from the local Quebec beer [1]. At that time, cobalt was added to beer as a foam stabiliser and was also used medically in tablet form for the treatment of anaemia [2]. Currently, it is widely used in metallurgy; human exposure can result from alimentary, occupational, therapeutic, and unintended iatrogenic sources, such as implants for hip replacements [2]. Drinking water contains approximately 2 *μ*g l^−1^, and average daily ingestion is 5–40 *μ*g in humans [3]. Cobalt is an essential trace element and is found in vitamin B 12 (cobalamin). Under physiological conditions, the nutritional intake is balanced by renal excretion [4]. High plasma levels, however, may interfere with aerobic cellular respiration, intracellular Ca^2++^ regulation, and can cause DNA damage, resulting in widespread systemic effects, principally affecting the circulatory, thyroid endocrine, haematological, and neurological systems [2, 5]. CMP following hip arthroplasty has received increasing attention, and there are a growing number of case reports with 23 cited in a recent review in 2018 [6]. Toxicity, alongside a high rate of implant failure with the need for revision surgery and adverse local tissue reactions, has contributed to the waning popularity of metal-on-metal (MoM) prostheses. According to recent data from the UK joint registry, less than 1% of primary hip replacements use MoM implants [7]. In this study, we describe the case of a patient receiving a heart transplant due to CMP following bilateral MoM hip arthroplasties.

## 2. Case Presentation

The patient underwent sequential left and right DePuy (DePuy Synthes; Raynham, MA, U.S.) MoM hip replacements for osteoarthritis in the United States in 2009 and 2011, respectively. His postoperative course was unremarkable. After relocating to the UK in 2017, he developed fatigue, exertional dyspnoea, and exercise intolerance. Echocardiography revealed a large pericardial effusion, which required drainage on three occasions. He was referred for specialist assessment. Simultaneous left and right heart catheterisation was performed, and there was no haemodynamic evidence of pericardial constriction. Cardiac MRI showed severe impairment of left and right ventricular systolic function, with extensive late gadolinium enhancement (myocardial scar). Endomyocardial biopsy was performed, but this did not yield a histological diagnosis. Genetic screening did not identify any variants associated with dilated cardiomyopathy. The cause of his cardiac problems was uncertain.

In August 2019, the patient was admitted to the intensive care unit (ICU) with decompensated heart failure, acute kidney injury, new onset atrial fibrillation, and a recurrent pericardial effusion. A repeat endomyocardial biopsy showed myocyte hypertrophy and fibrosis. He was treated empirically with corticosteroid and colchicine for what was assumed to be inflammatory myopericarditis. He continued to deteriorate and was referred for heart transplant assessment. At the same time, he was also referred to a rheumatologist with regard to the possibility of an underlying autoimmune condition.

In early 2020, his rheumatologist suspected cobalt-chromium toxicity after taking a detailed history. Serum cobalt and chromium levels were elevated at 5647.60 nmol l^−1^ and 1279.20 nmol l^−1^, respectively (normal: cobalt <10 nmol l^−1^; chromium<40 nmol l^−1^). Subsequent reanalysis of the myocardial biopsy specimens showed elevated cobalt (4.70 *μ*g.g^−1^) and chromium (17.20 *μ*g.g^−1^) levels in the myocardial tissue, confirming a diagnosis of cobalt cardiomyopathy. He did not have any evidence of neurological, liver, or thyroid involvement, and his symptoms were principally that of congestive cardiac failure.

The risks and benefits of heart transplantation were discussed. Redo hip replacement in order to remove the cobalt-chromium alloy hip prostheses was suggested because of the possibility of recovery of heart function and, if heart transplantation was required, to eliminate the possibility of recurrent problems in the transplanted heart.

Revision arthroplasties were performed in sequence in August (left) and November (right) 2020. A combined general/regional anaesthesia technique with lumbar epidural was used. The patient was given tranexamic acid 15 mg/kg bolus followed by 10 mg/kg/h infusion to reduce blood loss and the risk of allogenic blood transfusion and subsequent sensitisation, which has the potential of making future donor organ matching more difficult. At each operation, there was evidence of periarticular metallosis and osteolytic lesions of the proximal femur and acetabulum before the original prosthesis was replaced for a metal-on-polyethylene version. He required elective intensive care admission following each procedure and was extubated within twelve hours of admission but required three days of inotrope support postoperatively.

He made good recovery from revision arthroplasty, but there was no improvement in heart function, and he remained in advanced heart failure with a left ventricular ejection fraction of 20% and severely impaired right ventricular function on echocardiogram. By February 2021, serum cobalt and chromium levels have decreased significantly to 202.9 nmol l^−1^ and 373 nmol l^−1^, respectively. He was placed on the routine heart transplant waiting list in March 2021, and a suitable donor organ was identified within one month. Our patient underwent cardiac transplantation under the authors' anaesthetic and medical care in our institution. The perioperative anaesthetic management conformed to our usual strategy for patients with severe cardiac dysfunction. The patient remained stable following induction of anaesthesia, and his intraoperative course was uneventful. Following a 4-day stay on ICU, he was transferred to the ward where he made an excellent recovery. There has been no histological evidence of cobalt toxicity in the posttransplant biopsies so far.

## 3. Discussion

Cobalt-chromium alloys are used widely in medical implants, such as stents, dental implants, and various joint replacements [2]. Although current joint prostheses mostly use metal-on-polyethylene implants, it is estimated that approximately 1 million MoM hip arthroplasties have been performed in the US alone and around 60,000 in the UK [5, 7]. In 2011, the US Food and Drug Administration mandated postmarket surveillance of all MoM hip systems and both the American Academy of Orthopaedic Surgeons and the Medicines and Healthcare Products Regulatory Agency (MHRA) produced guidelines for follow-up and management. The MHRA recommended annual clinical, radiological, and laboratory assessments [5, 8]. In a recent systematic review of systemic cobaltism, 21 out of 25 cases had hip joint symptoms (such as pain or swelling) at the time of diagnosis, and all had systemic involvement with the mean number of organ systems affected being 3.6 [8]. Constitutional and cardiovascular symptoms were most frequent, with many patients also complaining of neurological, thyroid, and haematological abnormalities [8].

Blood cobalt (B[Co]) levels correlated well with total symptom severity, and the mean B[Co] in this cohort was 324 *μ*g l^−1^ (5491 nmol l^−1^). Normal B[Co] is considered to be <0.6 *μ*g l^−1^ (or <10 nmol l^−1^), and according to the MHRA guidelines, levels above 7 *μ*g l^−1^ (7 ppb or 120 nmol l^−1^) indicate the need for annual follow-up and cross-sectional imaging of the joints [5, 8]. Management of implant-related cobalt toxicity consists of removal of the source and chelation therapy with N-acetylcysteine and/or ethylenediaminetetraacetate (EDTA) [5]. Both of these result in declining B[Co], but chelation without source control is not recommended, unless the patient is too unstable for further surgery [3, 6]. Following joint revision with or without chelation therapy, both neurological and cardiovascular symptoms tend to improve and may return to baseline, but some patients are left with significant organ dysfunction, such as the case we present [6].

Anaesthetists may encounter patients with unrecognised cobalt toxicity in both orthopaedic and nonorthopaedic surgery settings. The history of previous hip replacement with pain and/or swelling of the joint, combined with any of the symptoms given in Table 1, should raise suspicion and lead to discussion with the orthopaedic team in the first instance. The initial investigations for suspected implant-related cobalt toxicity include cross-sectional imaging of the joint and blood tests including blood cobalt levels, taken in EDTA sample tubes, and sent to a reference laboratory. If cardiovascular symptoms exist, early cardiology review with ECG and echocardiography is warranted.

The diagnosis of cobalt toxicity and CMP is challenging and requires a high index of suspicion. Results of B[Co] and imaging may take several weeks to become available. A formal diagnosis will be supported by blood and tissue cobalt levels, characteristic MRI findings, and the exclusion of other more common conditions following a specialist review. Given the limited number of cases in the literature, it is not possible to base perioperative recommendations on robust evidence; thus, a pragmatic approach should be taken.

Patients presenting preoperatively ahead of elective surgery with a history of previous hip replacement and symptoms suggestive of CMP should be referred for orthopaedic and cardiology review and be fully investigated before proceeding to surgery. This may present a golden opportunity to establish an early diagnosis and prevent perioperative and long-term complications. There is no evidence that delaying surgery while awaiting B[Co] levels is necessary or beneficial when faced with a similar patient presenting for an urgent or emergency intervention. Arrhythmias and circulatory instability are common, particularly in cases with severe premorbid cardiovascular symptoms, and it is advisable to use invasive monitoring throughout the perioperative period. It is difficult to make any recommendations about perioperative chelation therapy as elevated cobalt levels fall slowly and end-organ effects only recover over a period of several weeks or months [5, 9].

Anaesthetists as perioperative physicians are optimally placed to identify patients with possible cobalt toxicity, allowing early diagnosis and potentially avoiding serious complications such as end-stage heart failure and need for a heart transplant like in the reported case.

## Figures and Tables

**Table 1:** Signs and symptoms of cobalt toxicity by organ systems.

Organ system							
Constitutional	Cardiovascular	Endocrine	Neurological	Hepatic	Gastrointestinal	Immune
Signs and symptoms
	Lethargy	Pericardial effusion	Hypothyroidism	Peripheral neuropathy (tremor, sensory deficit)	Hepatomegaly	Reflux oesophagitis	Enlarged mediastinal lymph nodes and lymph nodes around joint replacements
	Anorexia	Dyspnoea on exertion	Goitre	Hearing loss			
	Weight loss/difficulty gaining weight	Palpitations	Metabolic acidosis	Visual impairment			
	Polycythemia	Sinus tachycardia		Parkinsonism			
	Pyrexia of unknown origin	Hypotension		Cognitive decline			
		Cyanosis					
		Low voltage on ECG					
		Reduced ejection fraction					
		Elevated serum enzymes (raised troponin and BNP)					
		Elevated myocardial cobalt levels					
		Elevated serum cobalt levels					
		Absence of ischemia and other common causes of cardiomyopathy					

Adapted from [5, 8].
